# The Ear

**Published:** 1892-02

**Authors:** 


### THE EAR.

The human ear is a much more delicate organ than most people suppose. It is extremely dangerous to interfere with it by use of ear-picks, or any of the various instruments used for the purpose of cleaning it from wax. The wax is a natural secretion, and unless the ear becomes diseased it does not accumulate any faster than is necessary to protect the passage from the entrance of insects and various particles which might otherwise be forced in and tend to interfere permanently with the hearing. The greatest care is necessary in washing the ears of little children. They should be washed outside, but on the inside only so far as the finger wrapped in a soft towel will go. The practice of forcing a hair pin or any other hard instrument into the ear passage is fraught with danger of injuring the membrane and causing permanent deafness. Earache is a malady of childhood and causes most distressing pain. The simplest remedy for it is to take a little cotton dipped in warm sweet oil and put it in the ear passage. A danger that may arise from doing so simple a thing as this is that minute particles of the cotton may be left in the ear. To prevent this, some physicians advise making a little wad of the cotton and wrapping it in the finest and thinnest linen cambric that can be found, and dipping this in warm sweet oil. In case of intense pain, a few drops of hot laudanum or camphor may be used with the oil. When foreign bodies get into the ear they should be removed by syringing them out with warm water. To attempt to remove any thing from the ear passage by forcing an instrument in is a rash thing for any one except an aurist to undertake. The best medical practitioners refuse to treat affections of the ear or eye, but send their patients to specialists.

